# The use of circulating cathodic antigen rapid test and serology for diagnosis of active *Schistosoma mansoni* infection in migrants in Italy, a non-endemic country: a cross sectional study

**DOI:** 10.1590/0074-02760160355

**Published:** 2017-06

**Authors:** Laura Infurnari, Laura Galli, Alba Bigoloni, Alessia Carbone, Stefania Chiappetta, Angelo Sala, Norberto Ceserani, Adriano Lazzarin, Antonella Castagna, Giovanni Gaiera/

**Affiliations:** 1 San Raffaele Scientific Institute, Laboratory of Microbiology and Virology, Milan, Italy; 2 San Raffaele Scientific Institute, Infectious Diseases Department, Milan, Italy; 3 Saint Michel Centre, Society of Priests of the Sacred Heart of Betharram, Health Pastoral Diocese of Bouar, Bouar, Central African Republic; 4 Università Vita-Salute San Raffaele, Milan, Italy

**Keywords:** diagnosis, infectious diseases, international health, schistosomiasis, travel medicine, tropical medicine

## Abstract

Diagnosis of schistosomiasis in migrants coming from endemic areas can be difficult, especially in asymptomatic subjects. Light-intensity disease, in fact, may be missed due to the low sensitivity of the stool microscopy and serologic testing cannot distinguish between a resolved infection and an active infection in patients who have been infected and treated in the past, because specific antibodies can persist despite cure. We describe a cross-sectional study conducted on 82 migrants tested for *Schistosoma mansoni* on single blood (anti-schistosome antibodies, total IgE) and urine [point-of-care (POC) circulating-cathodic-antigen (CCA) test] samples. A positive POC-CCA test (active infection) resulted in two untreated patients with a positive serology while all patients (n = 66) with a past infection showed a negative POC-CCA test. POC-CCA urine test in combination with serology may be helpful in rapidly differentiate active from past *S. mansoni* infection in migrants coming from endemic areas.

*Schistosoma mansoni* is a parasite endemic in large parts of Africa (Hotez et al. 2014) and the reference standard for its diagnosis is stool microscopy (Kato-Katz or Ritchie tests) (Katz et al. 1972, Clerinx & van Gompel 2011, Gomes et al. 2014). The sensitivity of stool microscopy is, however, limited because of the number of eggs contained in each stool sample that can be reduced in light-infections (< 100 eggs/gram stool) and that frequently change on a day-to-day basis; moreover, this test depends on operator-experience (Navaratnam et al. 2012, Utzinger et al. 2012, Erko et al. 2013, Ochodo et al. 2015). Usually, adult migrants with chronic *S. mansoni* infections have light-intensity disease that may be missed due to the unspecific clinical presentation, the small number of eliminated eggs and the low sensitivity of the most widely used diagnostic assays (Utzinger et al. 2012). Then, there is the need for more sensitive, user-friendly and rapid diagnostic tests. Many studies evaluated the applicability of circulating cathodic antigen (CCA) test for the diagnosis of *S. haematobium* with very low sensitivity and unsatisfactory results (Obeng et al. 2008, Midzi et al. 2009, Stothard et al. 2009). On the contrary, the use of CCA test in *S. mansoni* infections was highly sensitive, rapid and non-invasive. In particular, various studies conducted in endemic areas comparing the use of CCA test on urine to Kato-Katz technique on stools demonstrated a superior sensitivity of CCA test in detecting *S. mansoni* infections (Coulibaly et al. 2011, 2013, Shane et al. 2011, Navaratnam et al. 2012, Tchuenté et al. 2012, Colley et al. 2013, Erko et al. 2013, Adriko et al. 2014). According to the Ochodo et al.’s review (Ochodo et al. 2015), the sensitivity of CCA test was 89% (ranging from 62-97%) and the specificity was 55% (ranging from 27-84%); authors interpreted the values of specificity as the misdiagnosed infections of patients with light-infections (< 100 eggs/gram stool). The lack of specificity can be increased to 76% if CCA is combined with Kato-Katz (Ruganuza et al. 2015) or if CCA test is repeated on multiple urine samples (Erko et al. 2013). Sensitivity seems to be higher in lower transmission setting (Sousa-Figueiredo et al. 2013).

In western countries, the use of point-of-care-CCA (POC-CCA) is not yet standardised, even if there is some new example in the recent literature: Becker et al. (2015) described two cases in which CCA urine test was used to confirm diagnosis of *S. mansoni* infection in symptomatic subjects.

Here, we report the results of the application of a urine-based antigen detection test for rapid, non-invasive diagnosis of *S. mansoni* infection in asymptomatic migrants coming from endemic areas.

This is a cross-sectional study on 82 asymptomatic migrants, living in Italy and coming from Egypt, who participated to a survey for the assessment of schistosomiasis at the out clinic of Tropical Medicine of the Infectious Diseases Department, San Raffaele Hospital, since February 2014 to January 2016.

Subjects attending the Travel and Tropical Medicine Clinic for a post-travel (in an endemic area) visit were informed by a travel doctor about the purpose and procedures of this study; participation was voluntary and patients interested in participating the study provided their oral consent. All patients participating to the study were informed about the results of the serological and POC-CCA assessments. All patients with an active *S. mansoni* infection were treated with praziquantel (40 mg/kg/day in two doses for three days).

During consultation, patients were screened for the determination of anti-schistosome antibodies, by performing an enzyme immunoassay that uses purified *S. mansoni* antigen (NovaLisa *Schistosoma mansoni* IgG ELISA, NovaTec Immunodiagnostica GmbH), and total IgE; a single urine sample was collected and tested by using a POC assay to detect CCA for the diagnosis of *S. mansoni* infection (A&B Rapid Test Schistosomiasis CCA, Lucca Italy). A drop of urine and a drop of reagent were placed on a POC-CCA cassette; the results were read after 20 min and recorded as either negative, trace or positive according to the development of a coloured test band; a second band (control line) had to show up to make sure that the test was valid.

Results were scored as: negative if the control line developed but no pink test line appeared; trace if a light-pink test line appeared; positive if a dark-pink test line appeared and its colour may be equal or more intense than that of the control line. This classification system is similar to that adopted in other studies (Coulibaly et al. 2011, 2013, Shane et al. 2011, Stothard et al. 2011).

Travels and previous treatments for schistosomiasis were also investigated.

Total IgE were considered as elevated if values were above the upper normal limit (> 100 UI/mL).

An active infection to be treated was defined in presence of a single trace or positive POC-CCA result associated with positive anti-schistosome antibodies; a past infection with no need of treatment was defined by the presence of a treatment for schistosomiasis in the past or positive anti-schistosome antibodies associated with a single negative POC-CCA test; patients were defined as uninfected if both anti-schistosome antibodies and POC-CCA were negative and if they have never received a previous treatment for schistosomiasis.

The performance of the considered POC-CCA test was also evaluated in five healthy volunteer subjects never exposed to *Schistosoma* and in 10 symptomatic patients with an active schistosomiasis diagnosed in Saint Michel Centre (Central African Republic) by microscopic stool analysis. Healthy subjects and patients with active *S. mansoni* infections were assessed by anti-schistosome antibodies, and POC-CCA test as negative and positive controls.

Results were described as median [interquartile range (IQR)] or frequency (%) in relation to continuous or categorical variables, respectively. Data were analysed by use of the SAS Software, release 9.2 (SAS Institute, Cary, NC).

Overall 82 migrants were evaluated: 95% males, with a median (IQR) age of 43.8 (37.1-50.7) years. They lived in Italy since a median (IQR) of 17 (14.0-23.0) years and only 24/82 (29%) had a recent travel in endemic sites. Patients characteristics according to serology for *Schistosoma spp* are shown in Table.

**TABLE:** Patients characteristics according to serology for *Schistosoma* spp

Characteristic	Anti-schistosome antibodies Negative (n = 23)	Anti-schistosome antibodies Positive (n = 59)
Median age, years (IQR)	47.6 (34.1-55.5)	43.7 (37.4-48.8)
Male gender, n (%)	22 (96%)	57 (97%)
Nationality		
Egyptian	22 (96%)	59 (100%)
Median length of residence in Italy, years (IQR)	15 (11.5-24.5)	17 (14-22)
Travel in endemic area, n (%)		
≤ 12 months before study enrolment	4 (50%)	13 (77%)
> 12 months before study enrolment	4 (50%)	3 (23%)
Median IgE, UI/mL (IQR)	34 (0-166)	179 (49-571)
Elevated (> 100 UI/mL)	7 (30%)	38 (68%)
Urine POC-CCA test, n (%)		
Negative	23 (100%)	57 (97%)
Positive	0	2 (3%)
Treatment for *S. mansoni* in the past, n (%)	9 (39%)	46 (78%)
Months since last treatment	54.5 (33.0-89.2)	28.5 (15.5-56.4)

IQR: interquartile range; POC-CCA: point-of-care-circulating cathodic antigen.

Two male subjects (2.4%) without urinary symptoms had an active infection; 66 subjects (81%) had a past *S. mansoni* infection, while 14 people had neither an active nor a past infection.

None of the patients with an active infection and 55/66 patients with a past infection (nine patients with a negative serology and a negative POC-CCA test; 46 patients with a positive serology and a negative POC-CCA test) had been treated for schistosomiasis in the past; the median (IQR) time since the last previous treatment was of 31.2 (16.6-65.1) months.

Elevated total IgE were observed in one out of two patients with an active *S. mansoni* infection as well as in 41/66 (62%) patients with a past infection.

The two patients with an active infection were subsequently treated with praziquantel (40 mg/kg/day in two doses for three days); they were re-assessed after three months from the end of the treatment and a negative POC-CCA test was observed in both cases.

All the five healthy subjects were found to have negative serology and a negative POC-CCA test while positive serology and a positive POC-CCA test was observed in the 10 symptomatic patients with an active schistosomiasis.

Findings of this study seem to suggest that a positive POC-CCA test is likely associated with the presence of an active infection in patients with a positive serology, no previous treatment for schistosomiasis and regardless of total IgE values. In addition, a negative single POC-CCA test, regardless of total IgE values, may represent a sign of previous *S. mansoni* infection if associated with a positive serology (immunological memory) or a previous treatment. Such interpretation seems also supported by the good performance of the considered POC-CCA urine test as compared to microscopic examination in a small group of healthy volunteer subjects with negative serology never exposed to *Schistosoma* and of patients with an active schistosomiasis and positive serology. A negative POC-CCA test was found in all the healthy subjects while all patients with an active infection were CCA-positive.

Migration from Africa into Europe has greatly increased in recent years and European countries have to face with imported infectious diseases not typically present in Europe (de Laval et al. 2014). Serologic testing for anti-schistosome antibodies is indicated for diagnosis of travelers or immigrants from endemic areas who have not been treated for schistosomiasis in the past. In patients who have been infected and treated in the past, serologic testing cannot distinguish resolved infection from active infection because specific antibodies can persist despite cure (CDC 2016). The antigen test can detect active infection and its validity is supported by findings of previous studies showing that the positive and negative predictive values of a single POC-CCA test are 77% and 72-89%, respectively, if compared to multiple Kato-Katz thick smears as diagnostic reference standard (Erko et al. 2013, Becker et al. 2015).

As a strength of the study, we highlight that, to our knowledge, this is the first study assessing a sample of patients (although limited in number) living in non-endemic countries tested for schistosomiasis because of their origin instead of symptoms (Becker et al. 2015). Among the study limitations, we mention the small number of considered patients, likely affecting the estimates precision; for this reason, our findings may not be intended as confirmatory but rather exploratory. Another limitation is that no stool samples were available in the study patients, impairing the assessment of POC-CCA performance (i.e., sensitivity, specificity, positive and negative predictive value) as compared to microscopy, still representing the reference standard for schistosomiasis diagnosis. Further studies including this comparative assessment would be of great value.

Epidemiological surveys in large population of migrants to be tested with POC-CCA are warranted in order to prevent *S. mansoni* infection long-term complications and to reduce health care costs avoiding useless treatments in case of past *Schistosoma* infection (i.e. persistence of positive serology).

In conclusion, the reported data seem to suggest that the use of POC-CCA urine test may be a simple, non-invasive test to differentiate active from past *S. mansoni* infection and therefore rapidly guide treatment initiation in migrants from endemic areas and living in non-endemic countries. Further, larger studies, also providing a comparative assessment with stool microscopy, would certainly be of value in elucidating the potential usefulness and cost-effectiveness of POC-CCA test as a screening diagnostic tool also in asymptomatic migrants living in non-endemic high-income countries.
